# Innovative method for CO_2_ fixation and storage

**DOI:** 10.1038/s41598-022-05151-9

**Published:** 2022-02-01

**Authors:** Kenji Sorimachi

**Affiliations:** 1 Research Laboratory, Gunma Agriculture and Forest Development, Takasaki, Gunma 370-0854 Japan; 2 Present Address: Bioscience Laboratory, Environmental Engineering, Co., Ltd., 1-4-6 Higashi-Kaizawa, Takasaki, Gunma 370-0041 Japan

**Keywords:** Environmental chemistry, Environmental impact, Climate sciences, Environmental sciences, Chemistry

## Abstract

The concentration of CO_2_ in Earth’s atmosphere has been gradually increasing since the Industrial Revolution, primarily as a result of the use of fossil fuels as energy sources. Although coal and oil have been vital to the development of modern civilization, it is now recognized that atmospheric CO_2_ levels must be reduced to avoid the serious effects of climate change, including natural disasters. Consequently, there is currently significant interest in developing suitable methods for the fixation of CO_2_ in the air and in exhaust gases. The present work demonstrates a simple yet innovative approach to the chemical fixation of extremely low and very high CO_2_ concentrations in air, such as might result from industrial sources. This process is based on the use of aqueous solutions of the water-soluble compounds NaOH and CaCl_2_, which react with CO_2_ to produce the harmless solids CaCO_3_ (limestone) and NaCl (salt) via intermediates such as NaHCO_3_ and Na_2_CO_3_. The NaCl generated in this process can be converted back to NaOH via electrolysis, during which H_2_ (which can be used as a clean energy source) and Cl_2_ are produced simultaneously. Additionally, sea water contains both NaCl and CaCl_2_ and so could provide a ready supply of these two compounds. This system provides a safe, inexpensive approach to simultaneous CO_2_ fixation and storage.

## Introduction

Although Earth has undergone many periods of significant environmental change over time, the planet’s environment has been unusually stable for the past 10,000 years^1^. During this time, various natural systems regulated the Earth’s climate and maintained the conditions that enabled human development. However, these regulatory systems have been greatly disturbed, and the planet may be nearing a threshold beyond which unpredictable environmental changes may occur, such as increases in the mean global temperature^2^. To reduce atmospheric CO_2_ concentrations as a means of mitigating such effects, the so-called Paris Agreement was reached at the United Nations Climate Change Conference (COP20) in 2015. This agreement was based on the requirement to keep the increase in the mean global temperature below 2 °C relative to the temperature prior to the Industrial Revolution, and preferably less than 1.5 °C. At present, this goal is challenging based solely on the development of carbon-neutral energy systems. Even so, President Elect Joe Biden has stated that the United States of America will rejoin the Paris Agreement (rejoined historically today, January 20, 2021) and the current Prime Minister of Japan, Yoshihide Suga, has declared that Japan will achieve a carbon-neutral society by 2050. Additionally, the President of the People’s Republic of China, Xi Jinping, has declared that China will be carbon neutral by 2060. Even so, because the present atmospheric CO_2_ concentration is quite high, there are ongoing efforts to reduce the accumulated CO_2_ so as to prevent a climate change crisis. Climatologists have warned that a significant reduction in the level of CO_2_ in Earth’s atmosphere is required over the next decade^2^; therefore, it is necessary to immediately begin this process. The urgency of this work has been communicated by climate change activists such as Greta Thunberg, and “Fridays for Future” events have been held worldwide.

Although renewable energy sources, including solar radiation and wind, can result in reduced CO_2_ emissions, these alternative systems still require energy expenditure and may also involve CO_2_ production. Additionally, these renewable energy approaches do not remove CO_2_ that has already accumulated in the atmosphere, nor do they address the ongoing generation of CO_2_ from exhaust gases and industrial sources. Thus, even if a carbon-neutral society could be immediately achieved, the accumulated atmospheric CO_2_ would not be reduced. For these reasons, it is important to lower the CO_2_ level currently in Earth’s atmosphere and to develop practical means of doing so as soon as possible. For CO_2_ storage, geo-sequestration by injecting CO_2_ into underground geological formations, such as oil fields, gas fields, and saline formations, has been suggested^3,4^, although these systems are still projects for the future.

Plants consume large quantities of CO_2_ based on photosynthesis, in which CO_2_ and H_2_O are converted to carbohydrates using chlorophyll under sunlight. However, the planet’s largest forest, the Amazon, which greatly contributes to the removal of atmospheric CO_2_, is continually shrinking because of commercial development and serious fires. CO_2_ also dissolves in the oceans to form H_2_CO_3_, HCO_3_^−^ and CO_3_^2−^, and there is approximately 50 times as much carbon dissolved in the oceans as exists in the atmosphere^5^. Conversely, all living organisms produce CO_2_ during respiration, such that the rates of CO_2_ consumption and production were balanced before human activities produced huge amounts of CO_2_. Certain CO_2_ derivatives are used industrially^6^ and in medicine^7^. The synthesis of methanol from CO_2_ is particularly important because methanol is a primary raw material for the production of numerous other chemicals^8^. For example, our own group recently found that NaHCO_3_ and Na_2_CO_3_ accelerate glucose consumption in cultured cells^9,10^. These materials improve serum glucose levels in diabetes mellitus patients^11^. However, the rate of usage of CO_2_ compounds in such applications is obviously much smaller than the rate of CO_2_ production.

CaCO_3_ can be used as a component of concrete, and CO_2_ can also be reacted to generate important compounds such as methanol on an industrial scale^8^, although the CO_2_ must first be captured and concentrated or fixed in some manner. CaCO_3_ is also readily converted to CO_2_ by reaction with HCl and other acids. Additionally, it should be noted that large amounts of CaCO_3_ are produced naturally as coral or in the form of limestone.

CO_2_ can be captured from the ambient air or from flue gas via several techniques, including absorption^12^, adsorption^13–18^ and membrane gas separation^14,19^. Absorption with amines is currently the dominant technology, while membrane and adsorption processes are still in the developmental stages with the construction of primary pilot plants anticipated in the near future. Recently, it was reported that an amine compound, spiroaziridine oxindole, fixed efficiently CO_2_ under near ambient conditions and released CO_2_ under mild conditions^17^. However, to the best of our knowledge, these methods alone cannot achieve the necessary worldwide reductions in atmospheric CO_2_.

## Results and discussion

### CaCO_3_ precipitation

**Figure 1:**
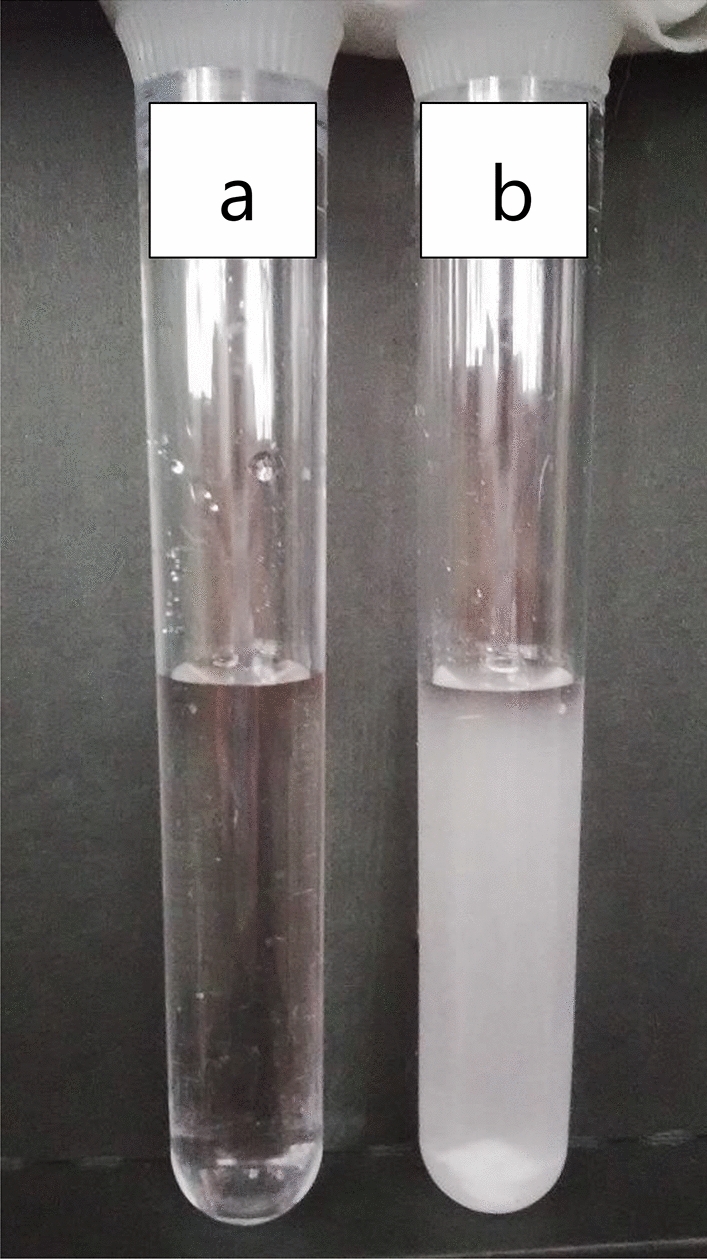
Photograph of CaCO_3_ precipitates. (**a**) A solution containing 0.05 N NaOH and 0.05 M CaCl_2_. (**b**) A solution treated with CO_2_ bubbles for 30 s at a flow rate of 2 cm^3^/s.

It is known that CO_2_ is absorbed by alkaline solution^16^. In the present work, CO_2_ was bubbled through an initially clear solution (Fig. 1a) containing 0.05 N NaOH and 0.05 M CaCl_2_ to form an immediate white precipitate (Fig. 1b).

$$2{\text{NaOH}} + {\text{CaCl}}_{2} + {\text{CO}}_{2} \to {\text{CaCO}}_{3} + {\text{H}}_{2} {\text{O}} + \, 2{\text{NaCl}}$$

**Figure 2:**
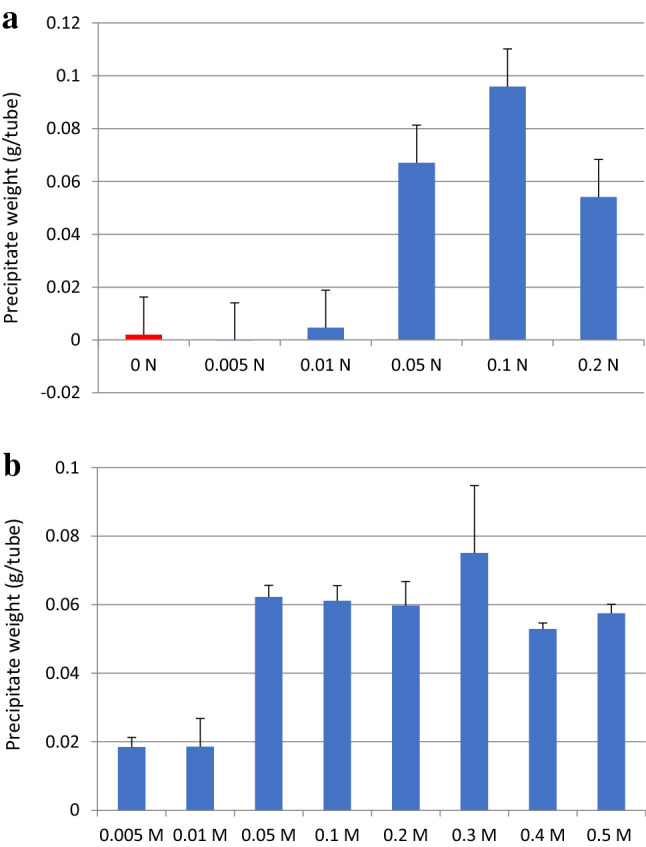
CaCO_3_ precipitates. (**a**) Quantities obtained from 3 mL of 0–0.4 N NaOH mixed with 3 mL of 0.1 M CaCl_2_ in a plastic tube followed by exposure to CO_2_ bubbles for 10 s at a CO_2_ flow rate of 2 cm^3^/s. (**b**) Quantities obtained from 3 mL of 0–1.0 M CaCl_2_ mixed with 3 mL of 0.1 N NaOH followed by centrifugation at 3000 rpm for 10 min (LCX-100, TOMY, Tokyo, Japan). Note that the final CaCl_2_ concentration was 0.5 M although the initial concentration was 1.0 M. The tube mass was determined before and after CO_2_ precipitation using an ME 204 instrument (METTLER TOLEDO). The vertical axis represents the mass of the wet precipitate and the plotted values are the mean plus or minus one standard deviation based on five replicates.

In other trials, varying the NaOH concentration between 0 and 0.5 N in the presence of 0.05 M CaCl_2_ was found to generate a white precipitate above 0.2 N NaOH even in the absence of CO_2_. Because this precipitate resulted from the formation of Ca(OH)_2_, the

$$2{\text{NaOH}} + {\text{CaCl}}_{2} \to {\text{Ca}}({\text{OH}})_{2} + 2{\text{NaCl}}$$

potential for CO_2_ incorporation in the form of CaCO_3_ was minimal under these conditions. Conversely, solutions with lower NaOH concentrations (from 0.05 to 0.1 N NaOH) together with 0.05 M CaCl_2_ remained clear, while the addition of CO_2_ bubbles produced a white precipitate (Fig. 2a). Under these conditions, CaCO_3_ precipitation occurred in the presence of CaCl_2_, which means that high NaOH concentrations were reduced by the formation of a Ca(OH)_2_ precipitate. However, prolonged bubbling with CO_2_ decomposed the CaCO_3_ precipitates to form Ca(HCO_3_)_2_, which is water soluble. As the concentration of CaCl_2_ was changed from 0 to 0.5 M, the amount of white precipitate was found to plateau at 0.05 M CaCl_2_ (Fig. 2b).

### One step CO_2_ fixation

**Figure 3:**
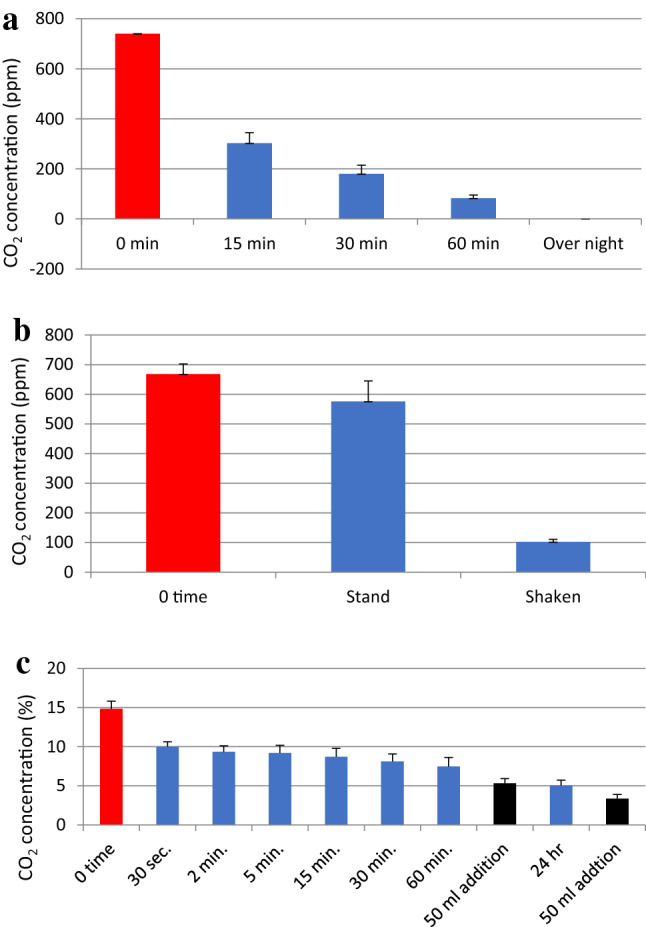
CO_2_ concentration changes in a bottle. (**a**) After the transfer of 10 mL of a solution containing 0.05 N NaOH and 0.05 M CaCl_2_ into a 2-L plastic PET bottle with a tight cap followed by standing for 15, 30 or 60 min. (**b**) After the transfer of 10 mL of this solution into a 1.4-L octagonal plastic bottle with a tight cap followed by standing or shaking for 5 min. (**c**) After the transfer of 50 mL of this solution into a 2-L plastic PET bottle with 15% CO_2_, followed by vigorous shaking for 30 s, then standing for various time spans. After 60 min, 50 mL of fresh solution was added with shaking for 30 s followed by standing for 24 h and shaking for 30 s. CO_2_ concentration in the gas phase was analyzed. All values are the means plus or minus one standard deviation based on four or five replicates.

The CO_2_ concentration in a 2-L bottle made of poly(ethylene terephthalate) (PET) was monitored to determine whether a solution containing 0.05 N NaOH and 0.05 M CaCl_2_ reduced the level of CO_2_. These trials showed that the CO_2_ reduction was clearly correlated with the time span over which the solution remained in the bottle and in contact with the internal atmosphere (Fig. 3a). Approximately 60% and 80% of the initial CO_2_ was removed after 15- and 60-min treatments, respectively. After allowing the plastic bottle to sit overnight, the CO_2_ in the bottle was completely removed. Thus, chemical fixation of CO_2_ emission, regardless of volume/concentration of CO_2_ could be efficiently captured and fixed by a solution containing 0.05 N NaOH and 0.05 M CaCl_2_. Laying the plastic bottle on its side increased the surface area of the solution and thus increased the CO_2_ removal rate (Fig. 3b).

At a high CO_2_ concentration of approximately 15%, the addition of 50 mL of a solution containing 0.05 N NaOH and 0.05 M CaCl_2_ followed by vigorous shaking of the 2-L bottle for 30 s by hand reduced the CO_2_ concentration to 10% (Fig. 3c). A further slight reduction of the CO_2_ concentration was obtained by subsequently allowing the bottle to stand. The addition of 50 mL of a fresh solution also resulted in an additional slight reduction and a further addition of fresh solution after 24 h again reduced the CO_2_ concentration (Fig. 3c). This slow reduction of the CO_2_ level after the initial rapid removal is attributed to the presence of insufficient quantities of NaOH and CaCl_2_. The pH of the solution after 24 h and following the third addition was 6.5, while that of the initial fresh solution was 12.19. These results indicate that the NaOH in the solution was completely consumed.

### Two steps CO_2_ fixation

In the above trials, a solution containing low concentrations of NaOH and CaCl_2_ was used in a one step process. When using high NaOH concentrations (above 0.2 N), the CO_2_ should first be treated solely with NaOH to prevent the formation of Ca(OH)_2_. This produces a solution of NaHCO_3_ and Na_2_CO_3_ to which CaCl_2_ can be added after reducing the NaOH concentration to less than 0.1 N. The latter method is based on two steps and allows the use of high concentrations of NaOH and CaCl_2_.

### Fog formation by absorbents

**Figure 4:**
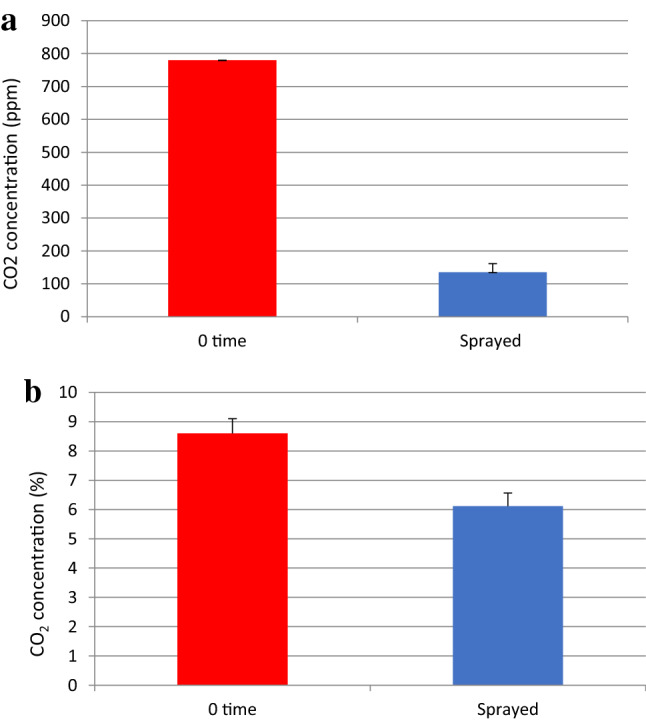
CO_2_ concentration changes obtained using a spray. A solution containing 0.05 N NaOH and 0.05 M CaCl_2_ was sprayed 10 times at 5-s intervals to provide a total volume of approximately 4 mL. (**a**) The solution was sprayed into a 2-L plastic PET bottle and (**b**) into a chimney model made from two milk boxes. In the latter case, the air and CO_2_ flow rates were 100 and 10 cm^3^/s, respectively. All values are the means plus or minus one standard deviation based on either six or ten replicates.

Because increasing the surface area of the highly concentrated NaOH solution is also important to ensuring efficient absorption of CO_2_, the generation of a fog can be beneficial. The formation of a fog greatly increases the liquid surface area and results in more rapid CO_2_ removal in the plastic bottle (Fig. 4a). In experiments using a chimney model, when the chimney contained high CO_2_ concentrations, the amounts of NaOH and CaCl_2_ in the solution were insufficient to react with all the CO_2_ at a gas flow rate of approximately 110 cm^3^/s (Fig. 4b). Thus, the solution could only capture a relatively small amount of the CO_2_ in the chimney model.

### Bubbling of CO_2_ gas

**Figure 5:**
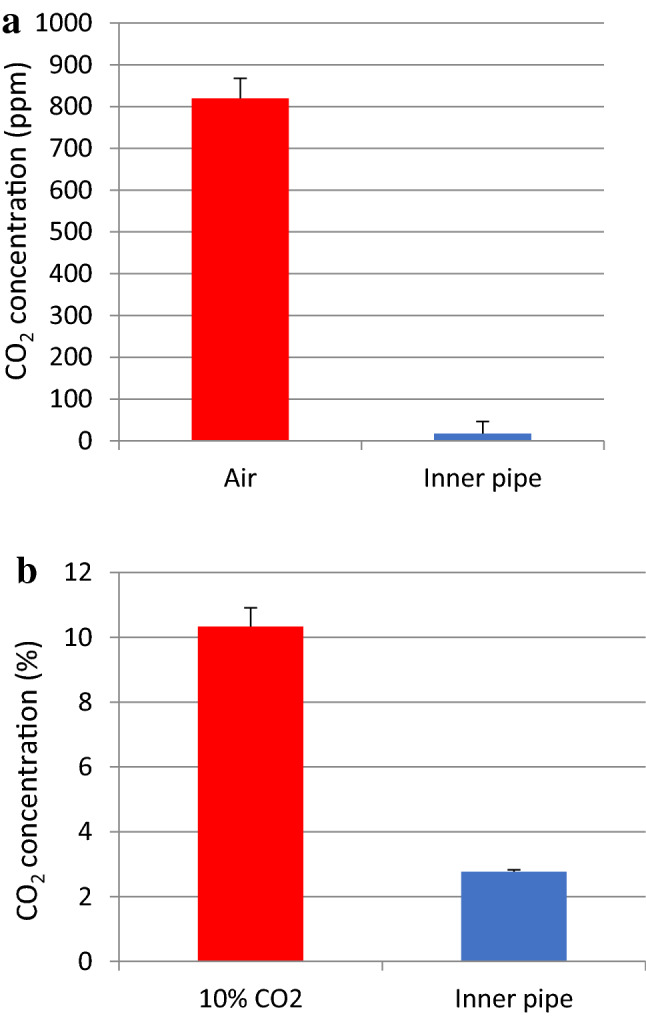
CO_2_ concentrations above the solution in the pipe apparatus when bubbling (**a**) air and (**b**) 10% CO_2_ in air through the solution. All values are the means plus or minus one standard deviation based on either nine (**a**) or three (**b**) replicates.

The area over which the reagent solution interacted with CO_2_ could also be increased by first passing the test gases through a porous stone to form bubbles. In these trials, a poly(vinyl chloride) pipe (40 mm in diameter and 50 cm in height) was partially filled with 250 mL each of aqueous solutions containing 0.1 N NaOH and 0.1 M CaCl_2_. Following this, the test gas was bubbled upwards through the solution at a flow rate of approximately 20 mL/s after passing through the porous stone at the bottom of the pipe. Under these conditions, the CO_2_ contained in the air was completely absorbed by the solution (Fig. 5a). In trials using this same apparatus with a very high CO_2_ concentration, the level was reduced from an initial value of 10–2.5% (Fig. 5b). These data indicate that this concept could be employed to reduce high CO_2_ levels in the exhaust streams from industrial operations such as thermal power plants and incinerators.

### Diagram showing the proposed CO_2_ fixation process

**Figure 6:**
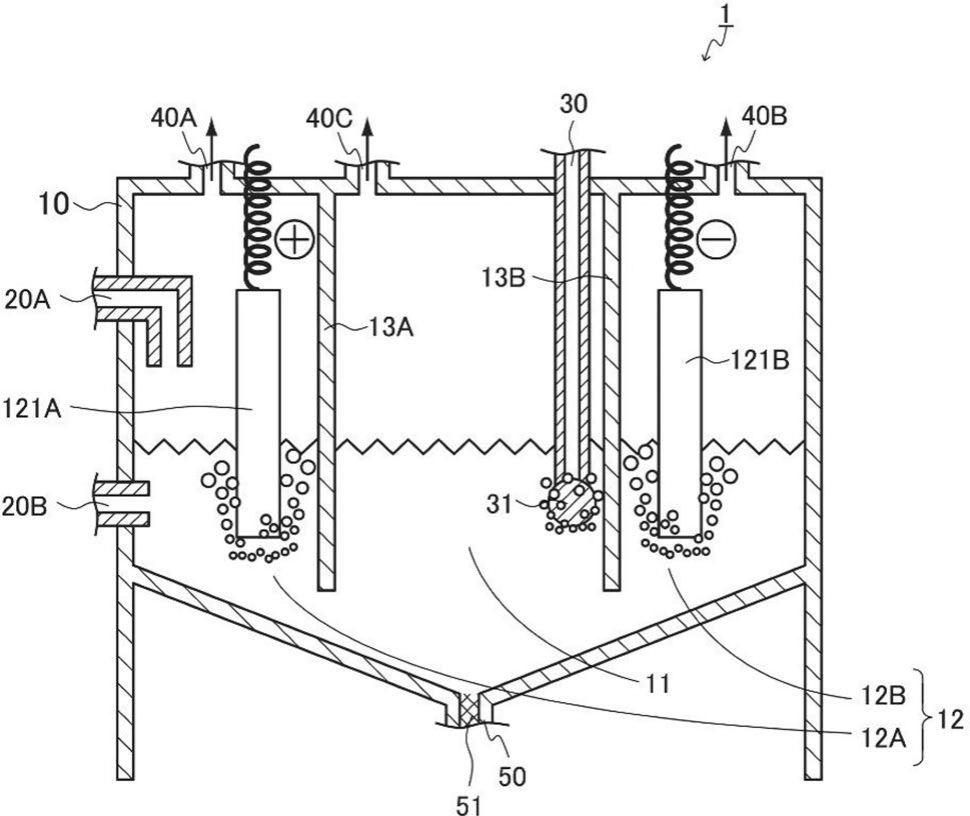
The figure shows proposed CO_2_ fixation process combined with the electrolysis of NaCl. 1: Carbon dioxide fixation apparatus, 10: reaction vessel, 11: reaction chamber, 12A: anode chamber, 12B: cathode chamber, 13A and 13B: partition wall, 20A and 20B: carbon dioxide fixing agent feeding units, 30: gas feeding unit, 31: insertion end point, 40A: Cl_2_ extraction portion, 40B: H_2_ extraction portion, 40C: air extraction portion, 50: liquid extraction portion, 51: filter, 121A: anode, and 121B: cathode. The original diagram was drawn by the author, and it was formally traced by Tsujimaru International Patent Office.

One means of producing NaOH on an industrial scale is the electrolysis of an aqueous NaCl solution. The products of this newly developed CO_2_ fixation system based on NaOH and CaCl_2_ are CaCO_3_ and NaCl, and this NaCl could therefore be subsequently converted to NaOH, H_2_ and Cl_2_ via an electrolytic process. Thus, CO_2_ could be captured using this system while simultaneously producing H_2_ and Cl_2_ (Fig. 6). Additionally, this process could potentially be integrated with existing generator systems based on atomic, thermal, solar, wind, hydro or wave power, and natural seawater could be used instead of an artificial NaCl solution in the electrolysis process.

**Figure 7:**
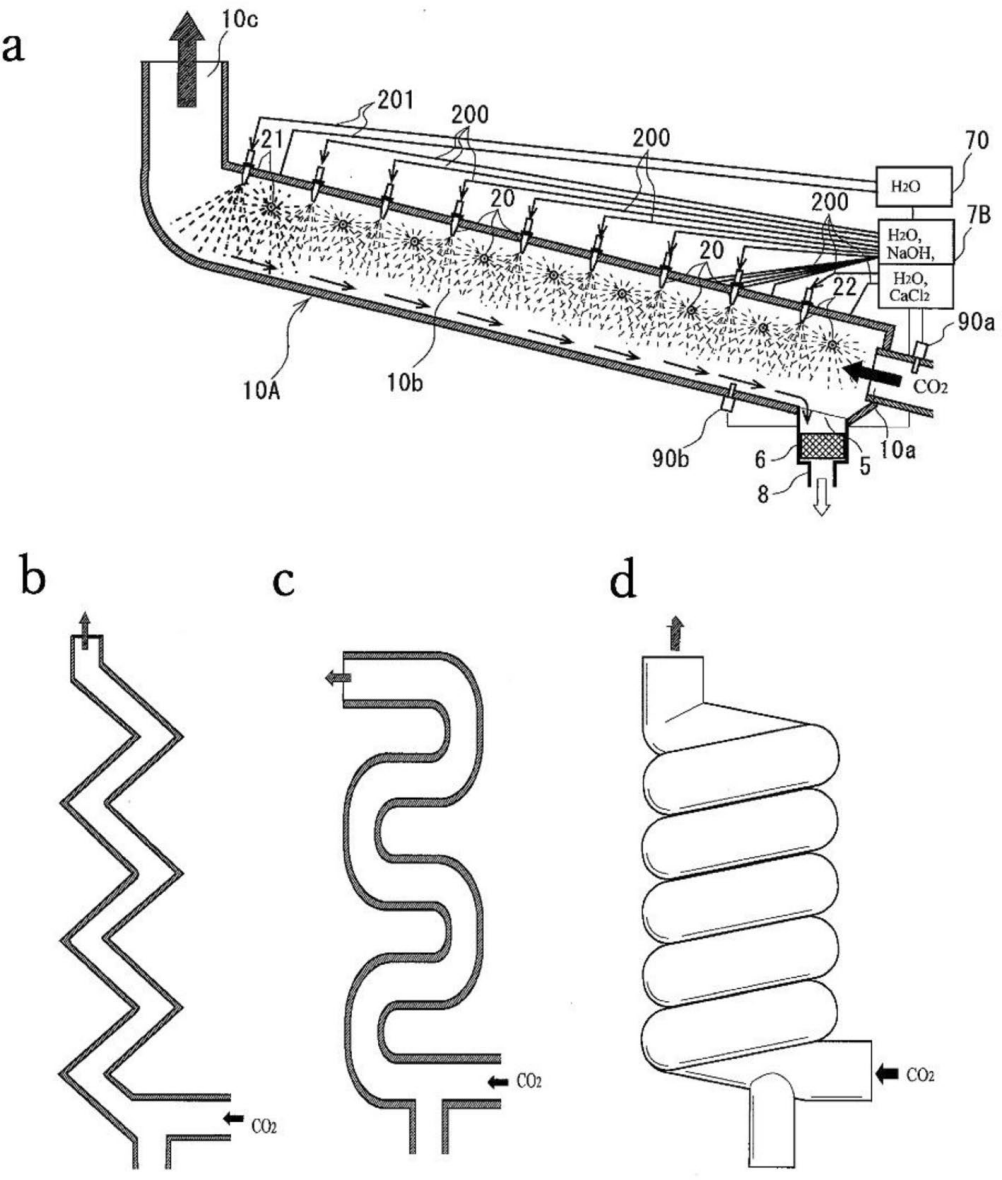
The figure shows proposed CO_2_ fixation process. The spray chamber could potentially have several different geometries, including (**a**) cylindrical, (**b**) zig-zag, (**c**) meandering, and (**d**) spiral. Legend: 5: exit for the CO_2_ fixation solution, 6: filter, 7A: fixation solution, 10A: reaction chamber, 10a: gas entrance, 10b: reaction chamber, 10c: exit, 20, 21 and 22: nozzles, 70: water tank, 90a and 90b: sensors, and 200 and 201: pipes. The original diagram was drawn by the author, and it was formally traced by Matsushima Patent Office, using software “Hanako” add in “Ichitaro”.

Conversely, the system presented in Fig. 6 is based on both CO_2_ fixation and NaCl electrolysis. Because the efficient absorption of CO_2_ with NaOH micro-droplets requires a large volume, while the electrolysis of a NaCl solution does not, a new CO_2_ capture plant design was developed, as shown In Fig. 7. This plant is intended to continually capture CO_2_ from the atmosphere or from exhaust gases. Using a large chamber equipped with spray nozzles, CO_2_ can be captured efficiently by droplets of the NaOH solution. As indicated in the figure, this chamber could have various geometries. The cylindrical and meandering shapes would be applicable to either reclining or standing structures, while the other morphologies would be suitable only for a standing structure. This system could also be combined with the NaOH generating process described in the preceding section.

Recently, plastic waste has been shown to be a significant environmental pollutant, and micro-plastics have been found to affect marine organisms^20^. A small portion of the plastics that are used daily in human activities are recycled, while the remainder is simply treated as waste. Many of these materials could be incinerated but instead are typically sent to landfills. However, if a simple method of fixing CO_2_ becomes available, this waste could be readily disposed of by burning without any environmental concerns and with the potential to generate energy. In addition, the current COVID-19 pandemic has resulted in vast quantities of waste materials potentially contaminated with the virus. It would be helpful to be able to burn contaminated plastic-based medical waste as a means of limiting the spread of infection. At present, chemical absorption using organic amines is typically employed to capture CO_2_ emitted from thermal power plants, but liberating CO_2_ from these complexes requires heat treatment that induces degradation. Because this treatment itself produces CO_2_, a new method that fixes CO_2_ would be highly beneficial. The present method employing inorganic compounds generates a stable product, based on the neutralization of NaOH along with the formation of CaCO_3_ and NaCl, both of which are harmless, stable natural compounds.

This technique is applicable to thermal power plants, chemical plants, large ships, combustion operations, incinerators and automobiles. Under strict regulations for air pollution, exhaust of oxide of nitrogen (NO_x_) and sulfur dioxide (SO_2_) which have great influence on environment and human health from coal combustion^21,22^ have been strongly prohibited by law. Contrary, there is no CO_2_ emission control, and this resulted in accumulation of atmospheric CO_2_ since the Industrial Revolution. Using this process, atmospheric CO_2_ can be spontaneously fixed based on a simple apparatus at various locations to generate CaCO_3_. This newly developed and facile system, which does not require organic chemicals, has minimal environmental impact and is completely sustainable, and so is expected to provide a means of reducing atmospheric CO_2_ levels so as to mitigate climate change. At present, there is worldwide recognition that climate change has become a crisis^2^. Because humans “who are the most evolved organisms”^23,24^ are responsible for this crisis, we have a moral duty to address the situation through global cooperation.

## Methods

### Chemicals

Reagent grade NaOH and CaCl_2_ were purchased from Wako-Junyaku Kogyo (Tokyo, Japan). Milli-Q water was used throughout the experiments.

### CO_2_ fixation

The reaction solution containing 0.05 N NaOH and 0.05 M CaCl_2_ was prepared in a commercial 2-L plastic PET bottle or a commercially available 1.4-L octagonal plastic bottle and the bottles were allowed to stand or were shaken for the stated periods.

In the fog trials, approximately 4 mL of the solution was sprayed into a 2-L plastic PET bottle, after which the CO_2_ concentration (in ppm) was measured using an RI-85 instrument (RIKEN). The chimney model was prepared by combining two 1-L paper milk boxes, after which air (at approximately 100 cm^3^/s) and CO_2_ (approximately 10 cm^3^/s) were supplied into the lower box. A layer of gauze was placed between the two boxes and approximately 4 mL of the solution was sprayed into the middle part of the lower box. The CO_2_ concentration (in %) was subsequently determined at the central point of the upper box using an XP-3140 instrument (COSMOS).
